# Correlation between preoperative ultrasonic features of medullary thyroid carcinoma and postoperative recurrence

**DOI:** 10.1186/s12885-021-07953-y

**Published:** 2021-04-01

**Authors:** Qiaodan Zhu, Dong Xu

**Affiliations:** 1 The Second Clinical Medical College of Zhejiang Chinese Medical University, Hangzhou, Zhejiang Province China; 2 Wenzhou Medical University, Wenzhou, 325600 Zhejiang Province China; 3 The Cancer Hospital of the University of Chinese Academy of Sciences (Zhejiang Cancer Hospital), No.1 East Banshan Road, Gongshu District, Hangzhou, 310022 Zhejiang Province China; 4 Institute of Basic Medicine and Cancer (IBMC), Chinese Academy Of Sciences, Hangzhou, 310022 Zhejiang Province China; 5 Key Laboratory of Head & Neck Cancer Translational Research of Zhejiang Province, Hangzhou, 310022 Zhejiang Province China; 6 Zhejiang Provincial Research Center for Cancer Intelligent Diagnosis and Molecular Technology, Hangzhou, 310022 Zhejiang Province China

**Keywords:** Medullary thyroid carcinoma, Ultrasound, Recurrence, Calcitonin

## Abstract

**Background:**

To investigate the factors that affect postoperative recurrence in medullary thyroid carcinoma (MTC) patients, including preoperative ultrasonic characteristics and other factors.

**Method:**

A retrospective analysis of 7 MTC patients who underwent the first thyroid surgery from 2009 to 2018 and who had complete follow-up data was conducted. According to the follow-up results, these patients were divided into the recurrence group (17 cases) and non-recurrence group (57 cases). The preoperative ultrasound characteristics, preoperative and postoperative calcitonin levels, and general informations of the two groups were recorded, respectively. Univariate and multivariate analyses were performed.

**Results:**

Single factor Kaplan-Meier (K-M) analysis showed that: ① Preoperative ultrasonic characteristics including tumor size > 40.0 mm, capsular invasion, and metastatic cervical lymph nodes, as well as preoperative calcitonin level > 565.8 pg/ml, and postoperative calcitonin (within one week) level > 45.0 pg/ml were positively correlated with the risk of postoperative recurrence of MTC (*P* < 0.05); ② There was no evidence to show that sex and age had a statistically significant effect on postoperative recurrence of MTC (*P* > 0.05). Multivariate Cox regression analysis showed that metastatic lymph nodes shown by ultrasound (HR = 5.368, 95%CI 1.063–27.104, *P* = 0.042) was an independent risk factor for postoperative recurrence of MTC.

**Conclusions:**

MTC patients with metastatic lymph nodes shown by ultrasound are prone to postoperative recurrence of MTC. In addition, MTC patients with a tumor > 40.0 mm, capsular invasion, preoperative calcitonin level > 565.8 pg/ml, and postoperative calcitonin level > 45.0 pg/ml are more likely to have postoperative recurrence.

## Background

Medullary thyroid carcinoma (MTC) originates from parafollicular cells, and its incidence is only 1–2% of all thyroid carcinomas [1]. Ultrasound is the first choice for the diagnosis of thyroid disease. MTC often shows preoperative ultrasonic characteristics such as solidity, low echo, calcification, and clear boundary [2, 3]. MTC is a moderately malignant tumor with a high recurrence rate (44.1–47.3%) [4, 5]. At present, researches on the factors affecting MTC recurrence have mostly focused on surgical methods, calcitonin (or procalcitonin), pathology, etc. [4–6], while researches on preoperative ultrasound characteristics of MTC are relatively rare. Therefore, this study attempted to determine the factors that affect postoperative recurrence of MTC based on preoperative ultrasonic characteristics, preoperative and postoperative calcitonin levels and general informations in MTC patients.

## Methods

### Clinical data

A retrospective analysis was conducted of 74 MTC patients who were admitted to hospital from 2009 to 2018 and had complete follow-up data. All patients underwent unilateral thyroid lobe plus isthmus excision or total thyroidectomy and central cervical lymph node dissection, and for those who had suspected lateral cervical lymph node metastasis by preoperative clinical and imaging evaluation, which was confirmed by fine needle biopsy, lateral cervical lymph node dissection was performed. All patients were confirmed to have MTC following pathological analysis. According to the follow-up results, the patients were divided into the recurrence group (17 cases, 23%, confirmed to have metastatic lymph nodes during second surgery) and the non-recurrence group (57 cases, 77%). Inclusion criteria were patients who had initial thyroid surgery in our hospital, complete follow-up data, preoperative ultrasonic examination, and postoperative pathological confirmation of MTC. Exclusion criteria were patients with a previous history of thyroid surgery for other thyroid diseases before MTC surgery, and those who died due to other diseases. Unilateral thyroid lobe plus isthmus excision or total thyroidectomy was performed in these patients.

### Methods

#### Examination methods

All 74 patients underwent preoperative ultrasonic examination, using ultrasound diagnostic equipment such as the Philips iU22 (L12–5 linear array probe, frequency 7.5–14 MHz), or the GE Logiq E9 (ML6–15 linear array probe, frequency 6–15 MHz). The patient was asked to place in the supine position, head back to expose the neck, scanned and the number and size of tumors, location, echo, composition, shape, boundary, aspect ratio, capsular invasion, calcification, blood flow and other data were recorded.

#### Image analysis

Two ultrasound attending physicians with more than 5 years of experience, analyzed the pre-stored images using the double-blind method. If the results were inconsistent, the ultrasound (associate) chief physician would be asked to assist in the analysis. Ultrasound image analysis included the number of thyroid tumors (single focus, multiple focus), size (defined by maximum length: ≤ 40.0 mm, > 40.0 mm), boundary (clear, unclear), composition (solid, cystic, cystic solid), morphology (regular, irregular), echo (very low echo, low echo, equal echo, high echo), aspect ratio (front and back diameter/left and right diameter ≥ 1, front and back diameter/left and right diameter <1), capsular invasion (yes, no), calcification (yes, no), blood flow (rich, none), metastatic cervical lymph node (the lymph nodes appear enlarged, round, etc.: yes, no). If multifocal MTC was present in patients, the largest MTC mass was selected for analysis.

#### Measurement of calcitonin

Preoperative calcitonin was measured in patients within 3 days before surgery, and postoperative calcitonin was measured within 7 days after surgery. Calcitonin levels were detected by electrochemiluminescence immunoassay (Elecsys Calcitonin: Roche Diagnostics GmbH. Penzberg, Bayern, Germany).

### Statistical analysis

SPSS25.0 statistical software was used for statistical analysis. Measurement data were expressed as mean ± standard deviation. The single factor Kaplan-Meier (K-M) method and log-rank method were used for survival analysis. Multiple factors were tested using Cox regression analysis. *P* < 0.05 was considered statistically significant.

## Results

A total of 74 MTC patients were followed-up from the date of diagnosis to November 30, 2019, with recurrence as the endpoint. Follow-up ranged from 14.5 to 128.9 months, with a median follow-up time of 42.0 months, and 17 patients (23%) relapsed during the follow-up period with an average survival time of 40.4 ± 3.2 months. The 1-, 3-, 5-, and 10-year overall survival rates were 91.9, 81.1, 78.4, and 77.0%, respectively.

### General information

A total of 74 MTC patients, including 42 males (56.8%) and 32 females (43.2%); aged 8 to 77 years, with an average age of 49.6 ± 1.6 years were enrolled in the study. According to the postoperative pathology results, 17 cases (23%) were included in the recurrence group and 57 cases (77%) were included in the non-recurrence group. The age (continuous variable) and recurrence (binary classification variable) were analyzed by the receiver operating characteristic (ROC) curve, and the optimal cut-off value was 52.50 years (area under curve (AUC) = 0.578, Sensitivity = 0.706, Specificity = 0.467). Therefore, the patients were divided according to age: ≤ 52.50 years old and > 52.50 years old. Single-factor K-M survival analysis showed that sex and age were not significantly associated with postoperative recurrence of MTC (*P* > 0.05). These results are shown in Table 1.

**Table 1:** Results of single factor K-M analysis related to recurrence of MTC

group	character	recurrence group (cases)	Non- recurrence group (cases)	Chi-square	*P*
Sex	male	12	30	1.542	0.214
	female	5	27		
Age	≤52.5	12	31	0.925	0.336
	>52.5	5	26		
Preoperative calcitonin(a*)	≤565.8 pg/ml	1	25	6.363	0.012
	>565.8 pg/ml	11	23		
Postoperative calcitonin(b*)	≤45.0 pg/ml	1	33	12.786	0.000349
	>45.0 pg/ml	15	21		
Tumor size	≤40.0 mm	13	53	5.837	0.016
	>40.0 mm	4	4		
Capsular invasion	no	1	29	9.273	0.002
	yes	16	28		
Metastatic cervical lymph nodes	no	2	31	8.442	0.004
	yes	15	26		

^*^Notes: a. 60 cases with preoperative calcitonin; b. 70 cases with postoperative calcitonin

### Calcitonin

Of the 74 patients, only 60 had data on preoperative calcitonin level and only 70 cases had data on postoperative calcitonin level. The normal reference range of calcitonin is 0–10 pg/ml. The range of calcitonin before surgery was 3.5–2000.0 pg/ml, and the range of calcitonin after surgery was 0.5–2000.0 pg/ml. Preoperative calcitonin was normal in 2 cases and elevated in 58 cases. Postoperative calcitonin was normal in 18 cases and elevated in 52 cases. The preoperative calcitonin level (continuous variable) and recurrence (binary variable) were analyzed by the ROC curve, and the best diagnostic cutoff value was 565.8 pg/ml (AUC = 0.745, Sensitivity = 0.917, Specificity = 0.519). Preoperative calcitonin level was ≤565.8 pg/ml in 26 cases, and was > 565.8 pg/ml in 34 cases. The ROC curve analysis of postoperative calcitonin level (continuous variable) and recurrence (binary variable) was carried out, and the best diagnostic cut-off value was 45.045 pg/ml (AUC = 0.759, Sensitivity = 0.938, Specificity = 0.611). Among these patients 34 cases had postoperative calcitonin level ≤ 45.0 pg/ml, and 36 had levels > 45.0 pg/ml. Single factor K-M survival analysis showed that preoperative calcitonin level > 565.8 pg/ml and postoperative calcitonin level > 45.0 pg/ml were risk factors for the recurrence of MTC (*P* < 0.05). These results are shown in Table 1.

### Analysis of ultrasound characteristics

**Fig. 1:**
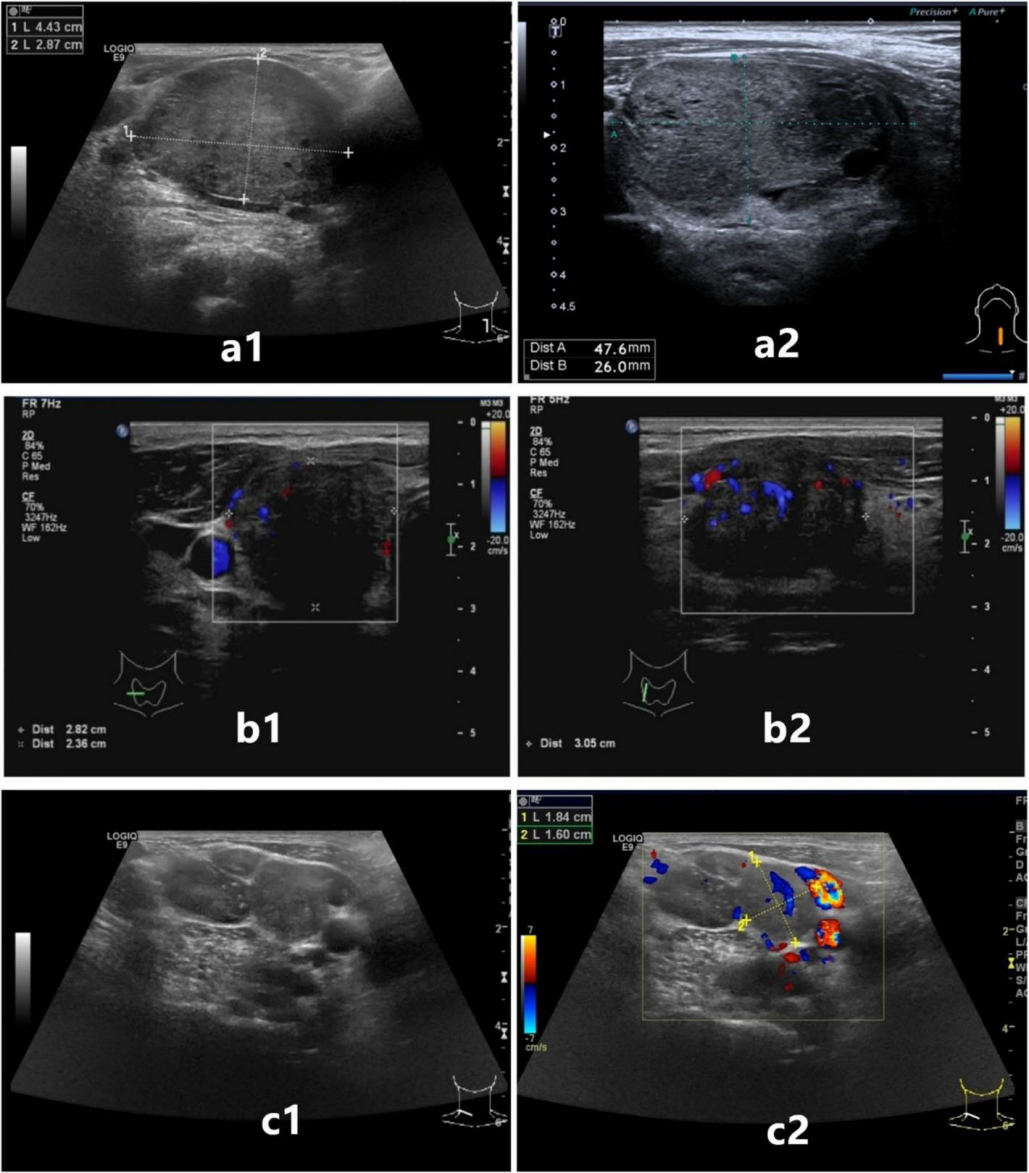
Ultrasonic characteristics of the MTC tumor: a1 and a2 show tumor size> 40.0 mm; b1 and b2 show capsular invasion; c1 and c2 show metastatic cervical lymph nodes: the lymph nodes appear enlarged, round

① In 17 cases in the recurrence group, the size of thyroid tumors was 8.0 to 61.0 mm as shown by ultrasound, with an average size of 28.18 ± 14.66 mm. ② In 57 cases in the non-recurrence group, the size of thyroid tumors, as shown by ultrasound, was 5.0 to 55.0 mm, with an average of 18.68 ± 11.57 mm. The tumors in these 74 patients were divided into ≤40.0 mm and > 40.0 mm with 40.0 mm as the boundary. In addition, the thyroid tumors in the recurrence group and the non-recurrence group were characterized by a single focus, solid, low echo, clear boundary, irregular morphology, aspect ratio (front and back diameter/ left and right diameter) < 1, calcification, and a blood flow signal. Single factor K-M survival analysis showed that a tumor > 40.0 mm, capsular invasion, and metastatic cervical lymph nodes were risk factors for the recurrence of MTC (*P* < 0.05). These results are shown in Table 1. The corresponding ultrasonic characteristics are shown in Fig. 1. However, there was no evidence to show that the number, composition, echo, boundary, morphology, aspect ratio, calcification and blood flow were significantly associated with the postoperative recurrence of MTC (*P* > 0.05) using single-factor K-M survival analysis. These results are shown in Table 2.

**Table 2:** Results of single factor K-M analysis of ultrasound characteristics and their relation to recurrence of MTC

group	character	recurrence group (cases)	Non- recurrence group (cases)	Chi-square	*P*
Number	single focus	10	42	0.847	0.357
	multiple focus	7	15		
Composition	solid	16	51	0.605	0.437
	cystic solid	1	6		
	cystic	0	0		
Echo	very low echo	3	17	1.119	0.773
	low echo	12	36		
	equal echo	2	3		
	high echo	0	1		
Boundary	clear	10	41	1.370	0.242
	unclear	7	16		
Morphology	regular	4	26	2.609	0.106
	irregular	13	31		
Aspect ratio (Front and back diameter/ Left and right diameter)	≥1	2	5	0.023	0.881
	<1	15	52		
Calcification	yes	12	36	0.525	0.469
	no	5	21		
Blood flow*	yes	12	38	0.337	0.562
	no	2	11		

*Notes: Only 63 patients had ultrasonic data on blood flow

### Multivariate cox regression analysis

Three ultrasonic characteristics were used to construct a multivariable Cox regression equation, including the size of the tumor, capsular invasion, and metastatic cervical lymph nodes. The results showed that metastatic cervical lymph nodes (Hazard Ratio (HR) = 5.368, 95% Confidence Interval (CI) 1.063–27.104, *P* = 0.042) were an independent risk factor for MTC recurrence. These results are shown in Table 3.

**Table 3:** Multivariate Cox regression analysis results

group*	B	Degree of freedom	P	HR	95%CI
Tumor size	0.853	1	0.153	2.347	0.728–7.570
Capsular invasion	2.181	1	0.050	8.855	0.996–78.730
Metastatic cervical lymph nodes	1.680	1	0.042	5.368	1.063–27.104

* Note: Tumor size (1 is ≤40.0 mm, 2 is > 40.0 mm), Capsular invasion (0 no, 1 yes), Metastatic cervical lymph nodes (0 no, 1 yes)

## Discussion

MTC accounts for an extremely small proportion of thyroid carcinomas, it is a moderately malignant carcinoma, and is derived from parafollicular cells. Its biological characteristics and degree of malignancy differ from other types of thyroid carcinoma derived from thyroid follicular cells. MTC is prone to recurrence after surgery. In this study, the recurrence rate after surgery was 23% (17/74), which was lower than the recurrence rate reported in previous studies (44.1–47.3%) [4, 5].

In terms of general information, Zhang Zaixing et al. [6] using univariate analysis found that sex (female) and age (≥45 years) were important factors affecting survival (recurrence, death, etc.) (*P* < 0.05). Using multivariate analysis, Hassan et al. [4] found that sex (male) was a poor prognostic factor (recurrence, death, etc.) in MTC patients (*P* < 0.05). In this study, the recurrence group and the non-recurrence group were more often male and young (≤ 52.5 years old), and there was no statistical significance between sex, age and recurrence (*P* > 0.05). The conclusions obtained in this study are partially consistent and partially contradictory with the conclusions of the above studies, which are related to the different definitions of the endpoints of these studies and the small number of cases.

As previously mentioned, MTC is derived from parafollicular cells that secrete calcitonin, and has the characteristic of elevated calcitonin. Some studies have found that patients with MTC who have elevated calcitonin after surgery were more prone to cervical lymph node metastasis and recurrence [7]. Using multivariate analysis, Hassan et al. [4] found that postoperative calcitonin doubling time in MTC patients was less than two years and that the rate of increase in calcitonin level was greater than 0.05 pg/ml/month and indicated a poor prognosis (*P* < 0.05). A meta-analysis showed that procalcitonin can be used as a marker to monitor recurrence during postoperative follow-up in MTC patients [5]. In this study, single factor KM survival analysis showed that preoperative calcitonin level > 565.8 pg/ml, and postoperative calcitonin level > 45.0 pg/ml were risk factors for the recurrence of MTC (*P* < 0.05, see Table 1). Only 60 cases had preoperative data on calcitonin level and only 70 cases had postoperative data on calcitonin level; thus, they were not included in the multivariate analysis. The conclusion that there was statistical significance between elevated calcitonin (or procalcitonin) and the recurrence of MTC patients is not new, and the specific values of calcitonin (or procalcitonin) that affect recurrence are not the same in each study. This is related to the limited number of cases enrolled in various studies, the large age span, and different test levels.

Starting from the ultrasonic features of MTC, research on the relationship between MTC and cervical lymph node metastasis is relatively extensive, while there are relatively few studies on the relationship between preoperative ultrasonic features and recurrence of MTC. Studies have shown that in sporadic MTC, single-factor chi-square analysis demonstrated that patients with tumors > 15 mm, irregular morphology, sharp edges, and masses located under the capsule have a higher risk of lateral neck lymph node metastasis (*P* < 0.05) [8]. The ultrasound characteristics of MTC include the size and number of tumors, echo, composition, boundary, morphology, invasion of the capsule, aspect ratio, calcification, blood flow, etc. In this study, single factor KM analysis showed that a tumor> 40.0 mm, capsular invasion, and metastatic cervical lymph nodes were risk factors affecting the recurrence of MTC (*P* < 0.05). These three factors represent the size of the tumor, the relationship with the surrounding adjacent tissues, and the status of the lymph nodes, which can be further applied to the preoperative T and N stages of MTC patients. Some studies have shown that T4 is a poor prognostic factor in multivariate analysis [4], and studies have shown that TNM staging affects prognosis in single factor analysis [6]. The results of the multivariate Cox regression analysis in this study showed that metastatic cervical lymph nodes were the only independent risk factor affecting the recurrence of MTC. In addition, this study found that in the recurrence group and the non-recurrence group, the tumors were mostly characterized by solidity, low echo, clear boundaries, and calcification. There were no statistically significant differences between the ultrasound features such as the composition, echo, boundary, calcification and recurrence of MTC.

In this study, the results of univariate KM analysis showed that: tumor size > 40.0 mm, capsular invasion, metastatic cervical lymph nodes, preoperative calcitonin > 565.8 pg/ml, and postoperative calcitonin > 45.0 pg/ml were risk factors for MTC recurrence (*P* < 0.05). The results of multivariate Cox regression analysis showed that metastatic cervical lymph nodes (HR = 5.368, 95%CI 1.063–27.104, *P* = 0.042) were independent risk factors for the recurrence of MTC. MTC patients with metastatic cervical lymph nodes are more likely to relapse. Although calcitonin was not included in the multivariate analysis, preoperative calcitonin > 565.8 pg/ml and postoperative calcitonin > 45.0 pg/ml also indicated the likelihood of relapse.

In this study, a total of 74 patients with MTC, including 17 cases with recurrence during follow-up and 57 cases with non-recurrence were included. Two patients who died due to other diseases were not included in the study. The findings of this study indicate that MTC patients with metastatic lymph nodes, as shown by ultrasound, are prone to postoperative recurrence of MTC. In addition, MTC patients with tumor size > 40.0 mm, capsular invasion, preoperative calcitonin level > 565.8 pg/ml, and postoperative calcitonin level > 45.0 pg/ml are more likely to have postoperative recurrence of MTC.

However, no other ultrasound features such as the composition, echo, boundary, and calcification were found to be related to MTC recurrence. In addition, the number of cases with MTC recurrence in this study did not reach half of the total number of cases, and the median survival time could not be determined in the K-M survival analysis. A future follow-up study is planned.

## Conclusions

MTC patients with metastatic lymph nodes shown by ultrasound are prone to postoperative recurrence of MTC. In addition, MTC patients with a tumor > 40.0 mm, capsular invasion, preoperative calcitonin level > 565.8 pg/ml, and postoperative calcitonin level > 45.0 pg/ml are more likely to have postoperative recurrence.

## Data Availability

The datasets used and/or analyzed during the current study are not publicly available due privacy rules but are available from the first author on reasonable request at the following email address zqd_whale@163.com

## Abbreviations

MTC: Medullary Thyroid Carcinoma
K-M: Kaplan-Meier
ROC: Receiver Operating Characteristic
AUC: Area Under Curve
HR: Hazard Ratio
CI: Confidence Interval
